# Engineering disease resistant plants through CRISPR-Cas9 technology

**DOI:** 10.1080/21645698.2020.1831729

**Published:** 2020-10-20

**Authors:** Swati Tyagi, Robin Kumar, Vivak Kumar, So Youn Won, Pratyoosh Shukla

**Affiliations:** a Genomic Division, National Institute of Agriculture Science, Rural Development Administration, Jeonju, Republic of Korea; b Department of Soil Science and Agricultural Chemistry, Acharya Narendra Dev University of Agriculture and Technology, Kumarganj, Ayodhya, India; c Department of Agriculture Engineering, Sardar Vallabhbhai Patel University of Agriculture and Technology, Meerut, India; d Enzyme Technology and Protein Bioinformatics Laboratory, Department of Microbiology, Maharshi Dayanand University, Rohtak, India

**Keywords:** Gene editing, CRISPR-Cas9, plant immune system, disease Resistance, susceptible genes, biotic stress

## Abstract

Plants are susceptible to phytopathogens, including bacteria, fungi, and viruses, which cause colossal financial shortfalls (pre- and post-harvest) and threaten global food safety. To combat with these phytopathogens, plant possesses two-layer of defense in the form of PAMP-triggered immunity (PTI), or Effectors-triggered immunity (ETI). The understanding of plant-molecular interactions and revolution of high-throughput molecular techniques have opened the door for innovations in developing pathogen-resistant plants. In this context, Clustered Regularly Interspaced Short Palindromic Repeats (CRISPR)-CRISPR-associated protein 9 (Cas9) has transformed genome editing (GE) technology and being harnessed for altering the traits. Here we have summarized the complexities of plant immune system and the use of CRISPR-Cas9 to edit the various components of plant immune system to acquire long-lasting resistance in plants against phytopathogens. This review also sheds the light on the limitations of CRISPR-Cas9 system, regulation of CRISPR-Cas9 edited crops and future prospective of this technology.

## Introduction

In nature, plants and microorganisms exist and evolve together.^1^ This interaction between plant and microbes is an integral part of their life, and based on the effect on the host, especially plants, this interaction (plant–microbe interaction) is defined either beneficial or harmful.^1^ Plants are susceptible to the number of plant pathogens, including bacteria, fungi, as well as viruses, which brings colossal financial shortfalls (pre- and post-harvest) to the farmers.^2^ In plants, biotic stress can impose more than 40% yield loss accounting for a total of 15% global food production drop.^3,4^ Furthermore, this yield loss put the most significant challenge against the world to feed its continually growing population, which is expected to be double by 2050.^3,4^

In past years, several chemicals in the form of insecticides, pesticides, or fertilizers have been used to minimize the yield loss and improve the plant health as well as develop resistance against pathogens.^5,6^ These chemical-based products can contaminate soil, water bodies, vegetation and impose risk to the host (consumer) as well as other organisms such as birds, fish, beneficial insects, and non-target plants.^5,6^ However, increasing awareness about the negative impact of chemical agents and development of rapid pathogen resistance pushed researchers to adopt the alternative techniques to combat it. New genomic approaches, such as new breeding techniques (NBT) and genetic engineering (GE) can alter the composition of plants and also boost resistance against microbial pathogens.^7^ GE, which is a biotechnological approach, can create stable, permanent, and heritable changes to the genetic code and can achieve specific potential goals, has several advantages over conventional breeding methods.^8^ For example, modification of plant traits such as yield, growth improvement, and to develop resistance against abiotic/biotic stresses via introducing, removing, or modifying the specific gene(s) is more straightforward and less laborious. It can be obtained in fewer generations using genetic engineering approaches than the traditional approaches.^9^ Another advantage of genetic engineering is the possibility of the interchange of the gene(s) among species and makes it a valuable tool to develop disease-resistant plants to meet the increasing food demand.^9^

More recently, CRISPR-Cas9 (clustered regularly interspaced short palindromic repeats)-Cas9 (CRISPR-associated protein) based tools have transformed agricultural science by showing its potential to edit the genome of plant species and providing new possibilities^.8,10,11^. CRISPR-Cas9 is approach is cost effective and user-friendly and thus becomes a trendy technique when compared with other GE techniques such as zinc-finger nucleases (ZFNs) and transcription activator-like effector nuclease (TALEN).^12–14^ A new protein is required every time following by its validation whenever there is a need to perform an experiment using ZFNs and TALEN and thus make it difficult as well as expensive to be used. However, CRISPR-Cas9, on the other hand, is relatively cheap and easy to be employed.^13^ CRISPR-Cas9 technology has been adopted widely to edit the genome of (more than 20 crops) plants for modifying several traits such as yield, growth improvement,^15^ and to develop resistance against abiotic/biotic stresses^16,17^ Here in this review, we will be discussing the use of CRISPR technology to develop resistance against a different class of pathogens by targeting the potential biomolecules involved in plant defense and develop disease-resistant plants.

## Mechanism of Plant Defense System

**Figure 1.**
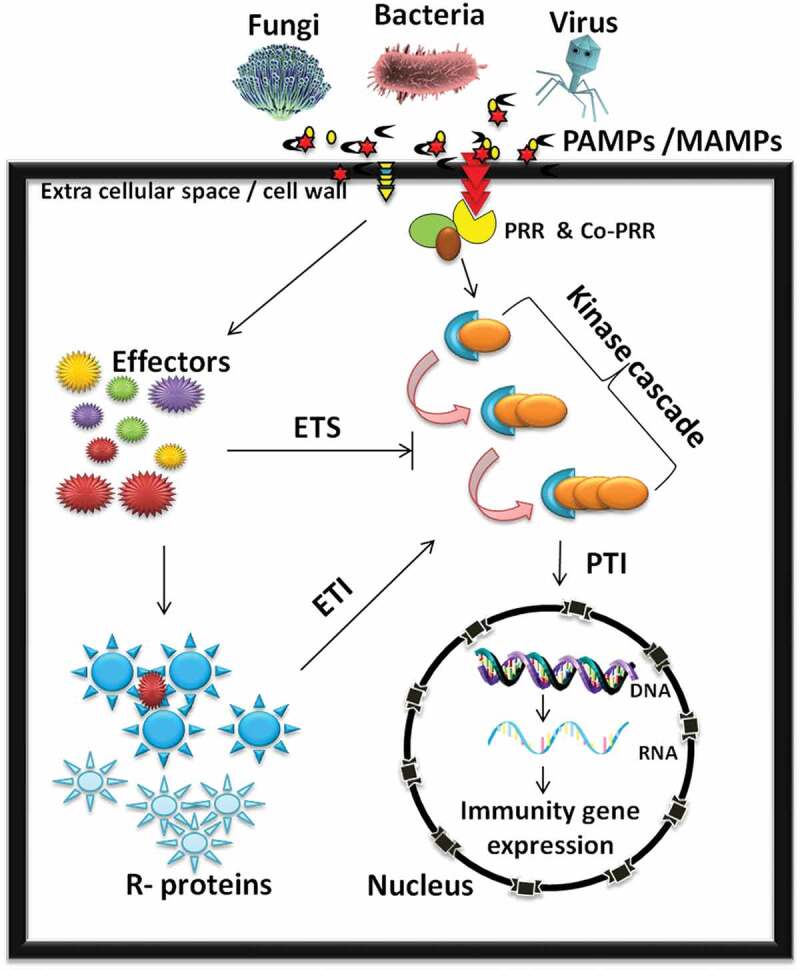
Graphical repersentation of plant defense system

In plants, the defense system starts with the cell to cell communication between a pathogen (or its component) and host plant (Fig. 1). During the plant–pathogen interaction, a complex series of events take place where several molecules from plant and pathogens are exchanged or interact.^18^ The type of biomolecules to be employed for defense is mostly depends upon the kind of interacting pathogen (bacteria, fungi, or viruses).^18^ For example, bacteria secrets virulence-related biomolecules through type II, III, and IV secretion systems^19^; and interact with host plants. On the other hand, fungi discharge several biomolecules (cell wall-degrading enzymes) to breach the first line of defense. These infectious biomolecules further initiate the infection or develop haustorium^20^ and affect the plant's health negatively. Contrary to it, viruses enter directly into the host through a mechanical injury or biological vector, and inject their genetic material and capsid proteins within the host and initiate infection.^18^

The schematic figure illustrates the intricate relations between plant innate immunity (PTI, ETI), ETS-related resistance response and the regulation of the genes involved in plant-pathogen interactions. On pthogen attack, plasma membrane-localized receptors (PRR) based on apthogen type such as bacteria, fungi or virus recognize the presence of PAMP’s or effectors in the extracellular environment and induce PAMPs Triggered Immunity (PTI), Effector Triggered immunity (ETI). Effector are any regulatory molecules secreted by pathogens which modifies the host protein to establish their growth and initiate Effecctor triggred susceptibility (ETS). R- ressistance proteins.

In general, plants possess a set of receptors called as trans-membrane protein recognition receptors (PRRs or wall-associated kinases abbreviated as WAKs).^21^ These receptors recognize the pathogen-associated or microbes–associated molecular patterns (PAMPs or MAMPs) and initiate a specific defense response known as PAMP-triggered immunity (PTI) to fight with the infectious pathogen.^5,22,23^ In response to this defense system, some pathogen secretes effectors to combat plant immunity, and suppress PTI by activating susceptibility (S) proteins and progress infection. This process is called as effectors-triggered susceptibility (ETS).^21^ Consequently, to battle with effectors, plants initiate the second line of defense employing resistance (R) genes, which are activated by recognizing the signals from effectors or avirulence (*avr*) proteins resulting in effectors-triggered immunity (ETI) . In general, PTI is a nonspecific form of immunity that is conserved across a class of pathogens and activated through the detection of PAMPs. Contrary to it, ETI is a highly specific type of immunity that is activated by the recognition of pathogen effectors and involves programmed cell death through hypersensitive response (HR) and checks pathogen growth at the infection site.^24^ PTI and ETI upon pathogen attack induce the expression of pathogenesis-related genes, alter a different kind of kinases such as mitogen-activated protein kinase (MAPKs) modify plant hormones, or transcription factors. It further affects the downstream events such as HR, reactive oxygen species (ROS) generation, cell-wall modification, stomata closure, or secretion of anti-microbial proteins and compounds, e.g., chitinases, protease inhibitors, defensins, and phytoalexins.^5,18,22^ Along with PTI and ETI, RNA interference (RNAi) is one another approach used by plants to detect and eradicate mugging viral pathogens.^7,25^

## Complexities of Disease Resistance in Plants

In nature, plants (as defending hosts) and pathogens (attacking parasite) have the constant race of arms to protect or kill each other. Thus, plants must adopt several defensive strategies against attacking pathogens (bacteria, fungi, and viruses) and vice versa. During evolution through selection pressure, plants come up with varied defense patterns and pathogens with elitist evasion abilities, as defined by Prichard and birch^26^ in the zig-zag model. For example, beside the physical barriers (which prevent the entry on pathogen within the cell), plants also use plasma membrane and inter/intracellular receptors that initiate safeguard strategies (as discussed above) once there is the perception of the pathogen itself or pathogen-derived modifications in host cells.^22^ Plants secrete some antimicrobial products that can recognize the pathogens or pathogen-derived compounds and can destroy it either by detoxification or inhibiting its virulence.^4^ Similarly, pathogens also have been evolved to overcome plant defense strategies and take over the plant system to initiate and spread infection. For example, several bacterial/fungal secrete cell-wall-degrading enzymes such as cellulase, xylanase, etc., that can breach the plant cell membrane protection and enable them to invade within the host.^27^ Some pathogens secret effector molecules that silence the host defense system and contribute to disease progression.^22,28^ Understandings of these molecular dialogs between plant and pathogens have provided an opportunity for genetic engineering researchers to develop disease-resistant or less susceptible plant varieties for crop development and sustainable agriculture.^29^

Advances in molecular biology techniques made it easier to modify the genome of a host either by introducing some genes those can breakdown the toxins, abolish the activity of cell wall-degrading enzyme and excrete antimicrobial compounds or deleting the genes those are susceptible to pathogen attack.^30^ The development of high-throughput molecular technologies made it easier to understand the mechanism of infection or immunity in plants or pathogens and providing opportunities to edit their genomes for developing resistance against plant pathogens. In this context, CRISPR based GE techniques are being utilized to alter the plant traits and improve pathogen resistance.

## CRISPR-Cas9 System as Bacterial Immune System and Magnificent Tool for Plant Editing

**Figure 2.**
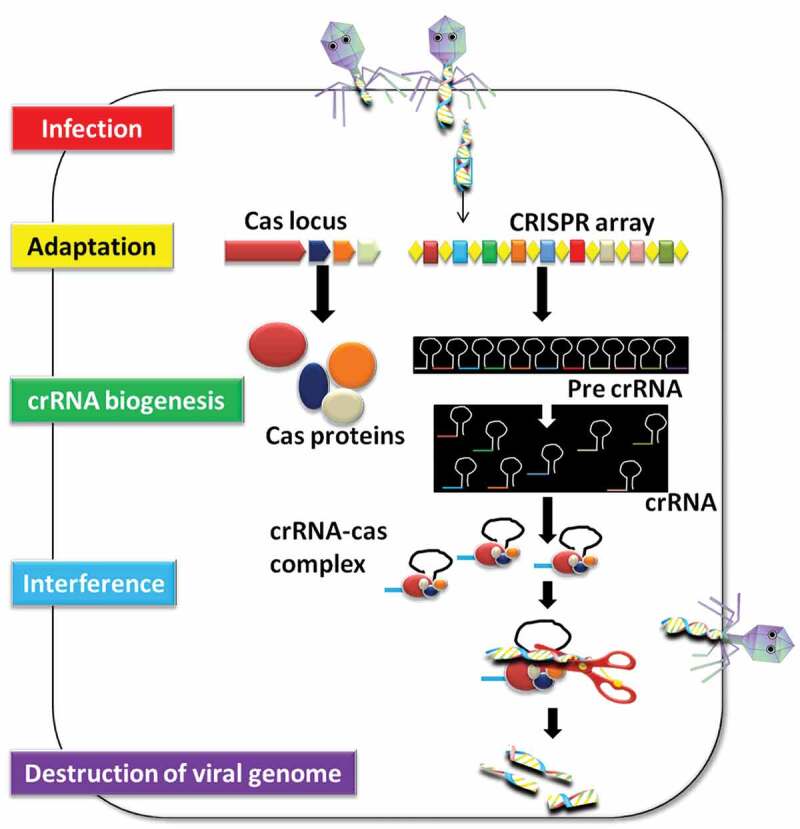
Graphical repersentation how CRISPR-Cas9 system work as bacterial immune system

**Figure 3.**
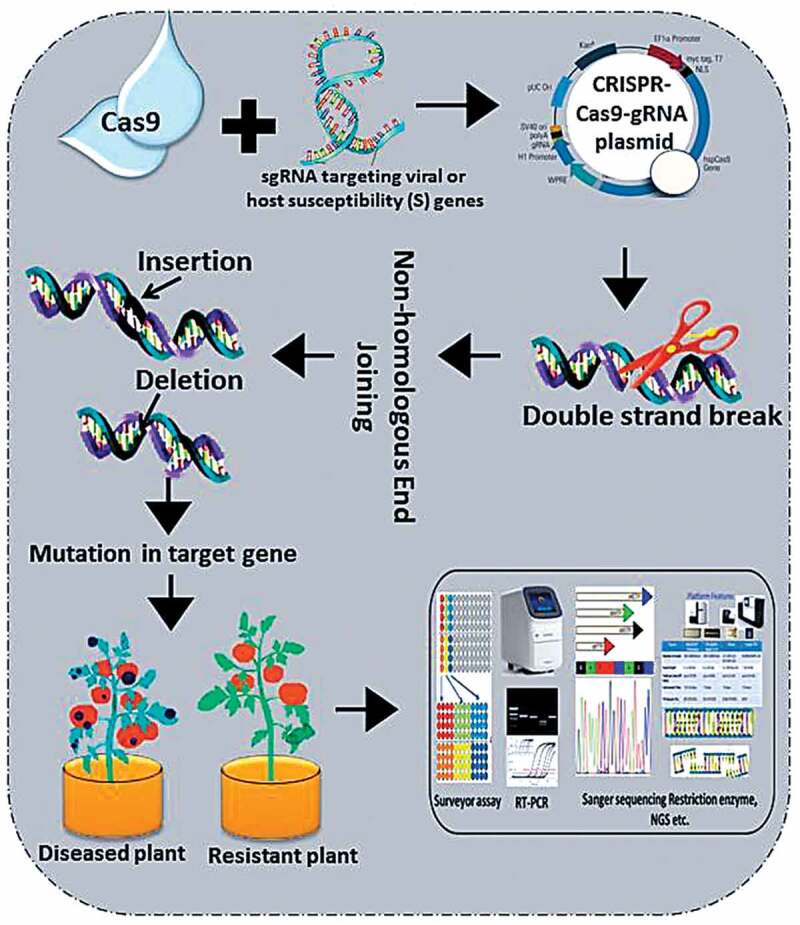
General work-flow of developing disease ressistance crops using CRISPR-Cas9 system

The CRISPR-Cas9 system is a RNA-mediated sequence-specific adaptive immune system of the prokaryotes, which provide protection against bacteriophages and viruses.^31^ This system has two arms: CRISPR array and Cas9 nucleases. CRISPR array is a sequence that comprises ~ 50 bp repeats separated by unique spacers of similar length. On the other hand, Cas9 is a CRISPR-associated nuclease protein which assists the foreign DNA degrading events.^32,33^ On pathogen attack, foreign (viral) genetic material is discovered, administered, and integrated into CRISPR array (Fig. 2). This episode leads to develop a kind of memory to the bacterial genome. Subsequently, CRISPR array is transcribed to form mature crRNA. This event is called as crRNA biogenesis. This crRNA further recognizes and degrade the viral DNA sequence harboring sequence complementary to the crRNA.^16,32,33^ This DNA degradation event is assisted by Cas9 nuclease, encoded by Cas genes flanking the CRISPR array.^34,35^ The ability of CRISPR-Cas9 system to generate double-strand break (DSB) in the genome, made it a wonderful tool to be applied as genomic scissors for genome editing.^36^ And, the execution of it in the laboratory does not require any debonair machinery except (i) a 18–22 bp long guide RNA sequence which is complementary to the target sequence (ii) and Cas9 nuclease to induce a DSB after protospacer adjacent motif (PAM) sequence.^3^ These CRISPR-originated DSB is repaired either by a low fidelity non-homologs end joining (NHEJ) process or a more specific Homology-directed repair (HDR) system.^37^ The error prone nature of NHEJ can incise the target sequence either by adding insertion or deletion (InDels) which result in loss of function and produce genetically modified crops (Fig. 3). After gRNA synthesis and PAM sequence identification, next is to clone the gRNA into desired vector, followed by transformation, screening, and validation.^38,39^ Till date, CRISPR-Cas9 has been used to manipulate the genome of almost every living system and being successfully exploited to edit the genome of several plants species stretching from monocot to dicots. The genome of model plants such as *Arabidopsis thaliana* and *Nicotiana benthamiana* as well as cash crops viz., rice, wheat, maize, barley, tomato, potato, cotton, soybean, citrus, grape, etc., have been modified despite their genome complexity in term of ploidy level.^16,40–46^

**Infection**. After infection, virus injedct its genome into the host, **Adaptaion** viral genome is acquired by CRISPR array (colored boxes; spacers and repeats) using Cas proteins; **crRNA biogenesis** Pre- Pre-crRNA is transcribed from the leader region by RNA polymerase and further cleaved into smaller crRNAs that contain a single spacer and a partial repeat by Cas proteins. **Interference**. crRNA containing a spacer that has a strong match to attacking virus genome initiates a cleavage event using Cas proteins are required for this process and interferes with virus replication.

Plant genome editing typically follow these steps: Finding a target gene and construction of artifical sgRNA, Vector construction follwoed by plant transformation. After transformation, sgRNA directs the Cas9 protein to bind target sequence and induce DSB. This DSB causes random mutations is repaired by error-prone HDR pathway based on the experimental objective. Plants with altered genome (with induced or deleted genes) is further screened and analysezed for desired trait. CRISPR, clustered regularly interspaced short palindromic repeat; Cas, CRISPR-associated; DSB, double-strand break; HDR, homology-directed repair; NHEJ, non-homologous end-joining; sgRNA, singleguide RNA.

## Fungal Resistance by Editing Host Susceptible Genes

Complicated lifestyle, genetic flexibility, and the ability to invade host easily make fungal plant pathogens a significant threat to agriculture.^47^ Several plant diseases such as rot, smut, rust, mildew, etc., are caused by fungal plant pathogens worldwide, which results in severe yield losses in pre-harvest and post-harvest crops.^5,7,47^ Along with the diseases, some fungal pathogens also produce secondary metabolites such as mycotoxins and epitomize serious health issues in animals as well as humans, which can be deadly sometimes.^47^ In plant-fungal interaction, ETI response is usually triggered by the interaction of pathogen effectors with plant R protein, which in turn activates the plant defense system. Traditionally, the introduction of the R gene into the plants was the most accessible and most promising approach to acquire resistance^48^ however, it did not prove durable due to species-specific nature of the pathogen. Moreover, the genetic flexibility of the fungi also enables them to disrupt the resistance (R- gene-mediated) by mutating the corresponding *avr* gene, which produces resistant pathogens and poses a significant challenge to control them.^49,50^ Thus, the resistance conferred by R gene is not long-lasting and can be breached easily. However, the modification of susceptible host genes (target genes, receptors involved in disease development) can be a practical and alternative approach to improve resistance. Also, loss of function mutation in S gene does not impact the overall plant health and developmental process. So, resistance against several fungal pathogens was developed by targeting the host susceptibility genes using CRISPR-Cas9 tools (Table 1).

**Table 1.** Plants edited to develop resistance against fungal pathogens

Plant species	Fungal pathogen	Disease	Target gene	Target Gene location	Target gene function	Method used	Achievements	Reference
*Oryza sativa*	*Magnaporthe grisea*,	Fungal blast	*OsMPK5*	Host S gene	Help in pathogen infection	Knockout	Showed potential of CRSIPR editing to improve disease resistance	^51^
	*Magnaporthe oryzae*	Rice blast	*OsERF922*	Host S gene	Help in pathogen infection	Knockout	Improved resistance to blast pathogen	^11^
	*Magnaporthe oryzae*	Rice blast	*ALB1, RSY1,*	Fungal	Required for pathogen growth	Site directed mutagenesis	RNP based CRISPR system to improve the resistance against pathogen and generation of marker free plants	^52^
	*Ustilaginoidea virens*	False smut	*USTA, UvSLT2*	Fungal	Required for pathogen growth	Gene replacement	Increased tolerance to hyperosmotic or oxidative stresses	^43^
*Triticum aestivum*	*Blumeriag aminis* *f. sp. tritici*	Powdery mildew	*MLO-A1, B1, and D1*	Host S gene	Help in pathogen infection	Knockout	Improved disease resistance	^53^
	*Blumeria graminis* *f. sp. tritici*	Powdery mildew	*TaEDR1*	Host S gene	Help in pathogen infection	Knockout	Improved disease resistance	^54^
								^44^
*Chardonnay*	*Uncinula necator*	Powdery mildew	*MLO-7*	Host S gene	Susceptible to pathogen	Knockout	RNP based system to enhanced disease resistance	^55^
	*Botrytis cinerea*	Gray mold	*WRKY52*	Host S gene	Host defense	Knockout	Enhanced resistance to fungal pathogen	^56^
*Golden delicious*	*Uncinula necator*	Powdery mildew	*MLO-7*	Host S gene	Susceptible to pathogen	Knockout	RNP based system to enhanced disease resistance	^55^
*Zea mays*	*Ustilago maydis*	Corn smut	*bW2, bE1*	Fungal	Effectors to start infection	Site directed mutagenesis	Investigated the virulence of pathogen	^57^
*Theobroma cacao*	*Phytophthora tropicalis*	Black pod disease	*TcNPR3*	Host defense gene	Host defense	Site directed mutagenesis	Improved disease resistance	^58^
*Solanum lycopersicum*	*Oidium neolycopersici*	Powdery mildew	*SlMlo1*	Host S gene	Help in pathogen infection	Knockout	Improved disease resistance	^59^
	*Fusarium oxysporum*	Wilt	*PKS4*	Fungal	Required for pathogen growth	Site directed mutagenesis	*PKS4* is responsible for bikaverin production and virulence	^60^
	*Phytophthora capsici*	Downy mildew	*DMR6*	Fungal	Required for resistance process	Site directed mutagenesis	Showed resistance against bacterial, fungal pathogens	^61^
	*Fusarium*	Wilt	*Solyc08g075770*	Host	Encodes a small transmembrane protein	Knock out	Showed resistance to fusarium wilt	^62^
	*Botrytis cinerea*	Gray mold	*SIMAPK3*	Fungal	Required for activation of PR related genes	Knock out	Showed improved resistance via reactive oxygen species accumulation and SA, JA defense pathways	^63^
*Glycine max*	*Phytophthora sojae*	Damping off	*Avr4/6*	Fungal	Effector proteins	Gene disruption/replacement	Showed increased resistance	^64^
*Brassica napus*	*Leptosphaeria maculans*	Blackleg disease	*HK*	Fungal	Provide pesticide resistance	Knockout	Increased resistance against pathogen	^48^

Fungi secrete cell wall-degrading enzymes to initiate the infection process, which loosens or degrades the plant cell wall and enables the pathogen to enter within the host. In response to this invasion by fungal pathogens, plants secrete inhibitors of these enzymes and add an extra layer of defense by other mechanisms *e.g*., by producing callose to strengthen the cell wall.^65^ The inhibitors of cell wall-degrading enzymes or genes involved in callose deposition can be a potent target for GE to develop resistance against fungi. The *Powdery Mildew Resistance 4* (*PMR4*) gene ortholog *SlPMR4*, which is involved in callose deposition (PRR gene), was targeted using CRISPR-Cas9 to genetically engineer the plants and improve resistance against powdery mildew pathogen *Oidium neolycopersici*.^65^ Interestingly, the loss of function of *PMR4* gene was due to inversion mutation, which improved the salicylic acid level and activated the HR response in plant upon pathogen challenge and conferred resistance. Thus, genetically engineered plants with inhibitors of cell-wall degrading enzymes, overexpressing PRR, or with impaired S genes can provide sustainable and improved resistance against pathogens.

The *mildew resistance locus O* (*MLO*) is a well-known host S gene in plant defense system. This gene encodes for a transmembrane protein (PRR) and studies conducted in the past and present with *MLO* editing confirm its role and ability to confer resistance as well as to exploitation in future.^66^ *MLO* gene was modified in three different crops *viz*. tomato, wheat, and grapevine using CRISPR-Cas9 to develop resistance against the powdery mildew disease caused by fungi and oomycetes.^53,55,59^ Homo-alleles of *MLO* in wheat plants -*TaMLO-A1, TaMLO-B1, TaMLO-D1* were mutagenized by CRISPR-Cas9 and interestingly, it was noted that *TaMLO-A1* mutated plants have developed resistance to *Blumeria graminis* f. sp. *tritici*, which cause powdery mildew disease.^53^ Similarly, grapevine gene *VvMLO7* was also mutated in protoplast culture using ribonucleoprotein (RNP)/CRISPR-Cas9 complex. However, the authors did not report the regeneration of plants using this CRISPR-Cas9 edited protoplasts but reported the improved fungal resistances in protoplast culture.^55^ Likewise, *the SlMlo1* gene in tomato was also truncated with CRISPR-Cas9 to generate resistant plants that were further self-pollinated to achieve CRISPR-Cas9-free plants without harming plant development and growth. The newly developed non-transgenic plant variety was named “Tomelo” by the authors and conferred complete resistance to *Oidium neolycopersici* causing Powdery mildew in tomato plants.^59^ Additionally, there was no report of “off-target” mutation after whole-genome Illumina sequencing outside the target region.^59^

Other susceptibility genes such as rice *Ethylene Response Factor 922* (*ERF922*) and *enhanced disease resistance 1* (*EDR1*), which are involved in ethylene signaling and pathogen resistance, respectively, was also targeted to genetically engineer the plants and improve their traits.^67^ Rice *OsERF922, OsSEC3A* was modulated with CRISPR-Cas9 and resulted in complete resistance against blast pathogen *M. oryzae* without disturbing the typical growth pattern of the plant.^11,68^ In another study, the null mutants of *Ossec3a* were found to have improved levels of salicylic acid (SA) along with the up-regulation of pathogenesis and SA signaling related genes. Though this genetic alteration resulted in dwarf plants when compared with wild type mutants; but showed resistance to the fungal pathogen.^68^ Mutation of one another host susceptible gene- *Taedr1* (an ortholog of EDR) in wheat resulted in improved resistance against *Erysiphe cichoracearum* powdery mildew pathogen. Genetic modification of this gene slightly influenced the plant growth but was found to have a broad range of resistance against bacteria, oomycetes, and other pathogens.^54^ Similarly, WRKY transcription factors that contribute to PTI and ETI actively and regulate defense response also another essential target gene to achieve resistance. The editing of the WRKY transcription factor in grapevine *VvWRKY52* also showed resistance to *Botrytis cinerea*. In this study, the transgenic plants carried mono and bi-allelic mutation and showed no remarkable difference in plant phenotype when compared with wild type plants.^56^ This study further showed the ability of CRISPR-Cas9-based tools to alter the genome of wood plants. In one another study, plant (*Theobroma cacao*) resistant to *Phytophthora tropicalis* was developed by transient leaf transformation. In this study, the potentiality of CRISPR-Cas9 was tested by pointing the *Non-Expressor of Pathogenesis-Related 3* (*NPR3*) gene and transgenic embryo further used to regenerate the plants which conferred resistance against *Phytophthora tropicalis*.^58^

Conclusively studies discussed here show the potential of the S gene in developing resistance against a broad range of pathogens. Generally, S genes are conserved in nature, and the discovery of one gene can enable the detection of more genes among or within the plant’s species. Resistance acquired by modification of susceptibility offers enormous opportunities in crop safety since a particular allele that confers susceptibility to one pathogen may confer resistance to another pathogen also.^69,70^ Collectively, these studies showed the potential of host S genes over R genes for developing pathogen resistance and pushed the necessities to find new S genes that can be exploited in future. However, side effects that appeared by editing/mutating the S gene should be considered. As stated above, S gene(s) is tightly linked with the plant growth and development and can cause pleiotropic effects.^68^ Hence these issues also should be taken into consideration while performing such gene(s) editing events in plants. Though few laboratory studies reported that genetic manipulation of these genes does not have any negative impact on plant growth and development,^32,71,72^ but durability and applicability of these plants at the field level is still needed to be determined.

## Bacterial Resistance by Editing Host Susceptible Genes

Bacteria are diverse, omnipresence, proliferate, and play a vital role (beneficial or harmful) in the living system.^9^ Phytopathogenic bacteria impose a significant threat to agriculture, cause several diseases such as spots, mosaics, rots and are hard to control, which result in severe yield loss.^73,74^ Borrelli et al.^66^ classified phytopathogenic bacteria as 1) crop-specific 2) polyphagous specific and 3) kingdom crosser. Specific bacteria produce specific disease symptoms in the specific host (for example, *Clavibacter michiganensis* cause bacterial ring rot disease in tomato) known as crop specific. In contrast, polyphagous-specific bacteria such as *Ralstonia solanacearum* produce multiple disease symptoms and have a broad host range and including monocot and dicots.^66^ On the other hand, kingdom crosser like *Dickeya dadantii* are those who are opportunistic pathogens and can cause several disease in different kingdoms also such as animals and plants.^66^ The pathogen of different categories imparts different responses in the host and thus put a great challenge to develop resistance. Constant evolving nature and horizontal gene transfer help bacteria to develop resistance against antibacterial products and spread infection.

In general, bacteria enter the cell either through natural means through plant openings such as stomata, stigma, etc., or mechanical wounds or by secreting bioactive molecules.^74^ The plant defense system recognizes these signals and responds appropriately to eliminate the pathogen (Fig. 1). However, successful pathogens use a complex signal cascade, and many host plant genes, including some S genes, also contribute to this, which helps pathogens to conquer the plant immunity and initiate infection.^7,8,47^ As discussed above, that achieved by the S gene may be more durable, so these genes are the potential targets to attain bacterial disease resistance via genome editing. However, there is not much improvement in the development of bacterial disease-resistant varieties. The reason seems that the genetic complexity of plants and the continuous evolution of bacteria in terms of bypassing the plant defense mechanisms. Still, there are fewer studies that have been conducted consuming CRISPR-Cas9 system to develop transgenic plants against bacterial pathogen than fungal and viral (Table 2).

**Table 2.** Plants edited to develop resistance against bacterial pathogens

Plant species	Bacterial Pathogen	Disease	Target gene	Target Gene location	Target gene function	Achievement	Method used	Reference
*Golden delicious*	*Erwinia amylovora*	Fire Blight	*DIPM-1, DIPM-2, DIPM-4*	Host S gene	Interact with pathogen effectors	RNP based system to enhanced disease resistance	Knockout	^55^
*Oryza sativa*	*Xanthomonas oryzae* pv. *oryzae*	Bacterial blight	*OsSWEET14, OsSWEET11*	Host S gene	Sucrose transporter gene	Demonstration successful editing of S genes	Knockout	^75^
	*Burkholderia glumae*	Bacterial blight	*OsMPK5*	Host S gene	Help in pathogen infection	Showed potential of CRSIPR editing to improve disease resistance	Knockout	^51^
*Solanum lycopersicum*	*Xanthomonas* spp	Bacterial blight	*SlDMR6-1*	Host S gene	Regulation of PR genes	Showed resistance against bacterial, fungal pathogens	Knockout	^61^
	*Pseudomonas syringae* pv. *tomato*	Bacterial blight	*SlDMR6-1*	Host S gene	Regulation of PR genes	Showed resistance against bacterial, fungal pathogens	Knockout	^61^
	*Pseudomonas syringae* pv. *tomato*	Bacterial Speck	*SlJAZ2*	Host S gene	Coronatine production	Improved resistance against bacterial speck disease	Knockout	^76^
*Citrus spp.*	*Xanthomonas citri* subsp.*citri*	Citrus canker	*CsLOB1*	Host S gene	Help in pathogen infection	Resistance to canker disease	Knockout	^77^
	*Xanthomonas citri* subsp.*citri*	Citrus canker	*CsLOB1*	Host S gene	Help in pathogen infection	Resistance to canker disease	Knockout	^78^

*Xanthomonas citri* is the causal agent of citrus canker disease in citrus plants and an economically important bacterial pathogen which causes severe yield losses.^78^ CRISPR-Cas9 system was used to achieve resistance against *X. citri* by modulating one of the target genes involved in ETI. The promoter (the effectors binding element (EBE) *PthA4*,) of *CsLOB1* (*lateral organ boundaries 1*) gene which was involved in host susceptibility, was mutated which in turn lost the ability to recognize and respond to bacterial effectors and hence showed increased resistance against infection.^77^ First Jia et al.^77^ edited the EBE*PthA4* element located in the promoter region of *CsLOB1*. They reported that the disease symptoms were reduced without affecting the plant phenotypes and did not produce any off-target mutation. Further this work was extended by Peng et al.^78^ who established the relationship between *CsLOB1* promoter and disease development in orange plants and confirmed that removal of *EBEPthA4* improved the resistance against *Xanthomonas citri* with no phenotypic changes. Similarly, tomato plants that are attacked by bacterial plant pathogens such as *Pseudomonas syringae, Xanthomonas* spp., and drop tomato production causing severe economic damage^79^; was also editing by CRISPR-Cas9 tools. It was noticed that infection with *P. syringae* pv. *tomato* improved the expression of *DMR6* (*downy mildew resistance 6*), act as a negative regulator to plant immunity and help to spread the infection.^80,81^ However, the deletion of *SlDMR6-1* (tomato orthologue) with CRISPR-Cas9 improved the resistance of tomato plants against a range of pathogens such as *P. syringae* pv. *tomato* and *Phytophthora capsici* and left no negative impact on plant health.^61^ Similar results were recorded when *DMR6* gene was mutated using CRISPR-Cas9 in *A. thaliana* plants; it not only improved the level of SA – a plant hormone involved in plant immunity but also provided resistance against a broad range of plant pathogens.^81^ Results obtained from such studies strengthen the idea of using genetic engineering to knockout or in a specific gene(s) and develop resistance against a broad range of plant pathogens. In another study, resistance against bacterial blight pathogen *Xanthomonas oryzae* pv. *oryzae* was attained by mutating the *OsSWEET13* – a susceptible host gene that is involved in sucrose transportation during pathogenesis, using CRISPR-Cas9.^82^ Expression of *OsSWEET13* is induced by the *X. oryzae* effector protein i.e., *PthXo2*, and thus boost the host susceptibility.^83^ Zhou et al.^83^ knocked out this *OsSWEET13* gene in rice and created that null mutant, which showed improved resistance against *X. oryzae*.

Similarly, *Jasmonate ZIM-domain-2* ortholog in tomato (*SlJAZ2*) was mutated by CRISPR-Cas9 to produce resistant plants against bacterial speck disease pathogen named *P. syringae* pv. *tomato*.^76^ After encountering the host plant, *P. syringae* secretes coronatine (*COR*), which is perceived by co-receptor *AtJAZ2*, and ultimately results in the stomatal opening, enabling bacteria to invade inside the plant and initiate infection. Mutation in *JAZ2* checks the stomata opening and provides resistance against bacterial speck disease.^76^ Generally, resistance against biotrophic pathogens brings susceptibility to necrotrophic pathogens and vice versa.^84^ Interestingly, in this case, the signals related to the jasmonic acid (JA) defense system remained out of stomata, so it did not have any effect on the SA defense system. Thus, *Sljaz21jas* mutant plants also remained unaffected to necrotrophic pathogen *Botrytis cinerea*, which causes tomato gray mold disease.^84^ This study thus provides a successful model of extrication of both (JA and SA) defense systems and gives an idea to target the host genome in such a way where it can provide resistance to a broad range of pathogens. Likewise, *Erwinia amylovora* produce a pathogen effector protein named as *DspE*. This *dspE* protein interact with *DspE-interacting proteins of Malus* (*DIPM*) genes- *DIPM 1, 2, 3, 4* and contribute to plant susceptibility for fire blight disease.^47,84^ These *DIPM* genes were truncated in apple protoplast using CRISPR-Cas9 to confer resistance against fire blight disease.^55^ Results from studies suggested that using CRISPR-Cas9 and S gene editing not only useful for developing resistance in crops with short life cycles but also very much useful in trees with longer life span. Furthermore, EvolvR- an advanced and modified form of CRISPR-Cas9 was used to create novel alleles in rice that can detect the full range of *Xanthomonas* strains and their ligands.^85^ Transgenic crops with novel alleles and resistant to broad pathogen range may prove a practical approach for sustainable agriculture. The future development of more specific tools that can easily and quickly identify the different pathogens and develop resistance in plants would extend its application and evolution of disease resistance in plants.

## Viral Resistance by Editing Host Susceptible Genes

Obligate parasitic nature, rapidly evolving genome, and high level of infectivity make plant viruses pose a serious threat to crop plants and drop the yield significantly.^47^ Based on genome type, the virus is classified as DNA viruses and RNA viruses. DNA viruses further divided into double-stranded DNA (dsDNA) and single-stranded (ssDNA) while RNA viruses have four more classes viz.- double-stranded (dsRNA) viruses, negative strand single-strand RNA (-ssRNA), positive-strand single-strand RNA (+ssRNA), and retroviruses. Among all these viruses, DNA viruses (family *Geminiviridae*) contain ssDNA, hijacks plant machinery, and replicate via rolling circle method, cause severe yield loss to several economically important crops such as cucumbers, tomato, potato, wheat, barley, etc. Viruses do not own translational machinery, so they take over the plant’s system, such as transcription/translational factors, and use it for their replication. Therefore, the active resistance against viruses (DNA or RNA viruses) can be achieved either by targeting the pathogenicity-related genes or by modifying the host susceptibility genes (Table 3).

**Table 3.** Plants edited to develop resistance against viral pathogens

Plant species	Viral Pathogen	Disease	Genome type	Target gene	Target Gene location	Target gene function	Achievement	Method used	Reference
*Arabidopsis thaliana*	*Turnip mosaic virus* (*TuMV*)	Mosaic	RNA	*eIF4E*	Plant (Host) S gene	Help in viral replication	Improved resistance without affecting the plant vigor	Knockout	^86^
	*Beet severe curly top virus* (*BSCTV*)	Curly leaf	DNA	IR, CP, Rep	Viral	Virus replication and assembly	Inhibit virus accumulation and provide resistant to virus infection	Site directed mutation	^87^
	*Tobacco mosaic virus* (*TMV*)	Mosaic	RNA	*ORF1a,ORFCP*, 3’- UTR	Viral	Virus replication	Improved virus resistance and inheritable mutation in plants	Site directed mutation	^88^
	*Clover yellow vein virus* (*CYVV*)	Necrosis	DNA	*eIFE14*	Plant (Host) S gene	Help in viral replication	Improved transgene‐free genetic resistance in plants	Site directed mutation	^89^
*Nicotiana tabacum*	*Tomato yellow leaf curl virus*; (*TYLCV), Beet curly top virus* (*BCTV), Merremia mosaic virus* (*MeMV*)	Curly leaf, Mosaic	DNA	Coding, noncoding region	Viral	Virus replication and assembly	Reduced accumulation of viral DNA and significantly attenuating symptoms of infection	Site directed mutation	^90^
	*Bean yellow dwarf virus* (*BeYDV*)	Dwarfness	DNA	Rep and intergenic sequence	Viral	Encode replication proteins	Reduced viral load and less infection symptoms	Site directed mutation	^91^
	*Beet severe curly top virus* (*BSCTV*)	Curly leaf	DNA	IR, CP, Rep	Viral	Virus replication and assembly	Inhibit virus accumulation and provide resistant to virus infection	Site directed mutation	^87^
	*Tobacco mosaic virus* (*TMV*)	Mosaic	RNA	*ORF1a, ORFCP, 3’- UTR*	Viral	Virus replication	Improved virus resistance and inheritable mutation in plants	Site directed mutation	^88^
	*Turnip mosaic virus* (*TuMV*)	Mosaic	RNA	TuMV-GFP, HC-Pro, coat protein	Viral	Virus replication and assembly	Provided RNA-guided immunity against RNA viruses	Site directed mutation	^28^
	*Cotton Leaf Curl Kokhran Virus* (*CLCuKoV), Tomato yellow leaf curl virus* (*TYLCV), Tomato yellow leaf curl Sardinia virus* (*TYLCSV), Merremia mosaic virus* (*MeMV), Beet curly top virus* (*BCTV*)	Curly leaf, Mosaic	DNA	IR, coat protein and Rep	Viral	Virus replication and assembly	Showed targeting noncoding and intergenic viral sequence can provide durable resistance	Site directed mutation	^92^
*Manihot esculenta*	*Cassava brown streak virus* (*CBSV*)	Leaf streak	RNA	*nCBP-1*&*nCBP-2*	Plant (Host) S gene	Help in viral replication	Suppressed disease symptoms	Knockout	^93^
*Hordeum vulgare*	*Wheat dwarf virus* (*WDV*)	Dwarfness	DNA	Rep, MP, LIR	Viral	Virus replication and assembly	Improved resistance against virus and reduced symptoms	Site directed mutation	^94^
*Musa spp.*	*Endogenous Banana streak virus* (*eBSV*)	Leaf streak	DNA	Non coding, coding regions	Viral	Virus replication and assembly	Showed potential of CRISPR system to achieve resistance against virus in banana plants	Site directed mutation	^95^
*Cucumis sativus*	*Cucumber vein yellowing virus* (*CCVYV), Zucchini yellow mosaic virus* (*ZYMV), Papaya ring spot mosaic virus-W* (*PRSV-W*)	Mosaic	RNA	*eIF4E*	Plant (Host) S gene	Help in viral replication	Enhanced resistance against several viral species	Knockout	^96^
*Oryza sativa*	*Rice tungro spherical virus* (*RTSV*)	Tungro	RNA	*eIF4G*	Plant (Host) S gene	Help in viral replication	Improved resistance against virus	Knockout	^97^

In recent years, different CRISPR-Cas9-based tools that employ *Cas9* or *Cas13a* nucleases have been used to engineer the plants genetically and develop resistance against plant DNA viruses.^90,91,98^ First report of using CRISPR-Cas9 based tools to develop resistance against DNA viruses came in 2015 from three different research groups. These groups developed resistance against geminiviruses such as *Beet severe curly top virus* (*BSCTV), tomato leaf curl virus* (*TYLCV), bean yellow drawf virus* (*BeYDV*) in model crops, e.g., *Arabidopsis thaliana, Nicotiana benthamiana*.^90,91,98^ Coding and non-coding parts of the viral genome such as capsid protein, replication protein was targeted with artificially created guide RNA (gRNA)/Cas9 nuclease proteins, which in turn resulted in fewer symptoms and improved viral resistance.^47^ Similarly, Ali et al.^92^ mutated the non-coding region of *Merremia mosaic virus* (MeMV), *Tomato yellow leaf curl virus* (*TYLCV), Cotton leaf curl Kokhran virus* (*CLCuKoV*) and *beet curly top virus* (*BCTV*) genome. The authors verified that the CRISPR tool is sufficient to induce targeted mutation, which makes virus frail to replicate and improve resistance in plants. This study revealed that the non-coding region of the viral genome resulted in the more resilient resistance than the coding sequences. The reason behind this observation was the quickly evolving nature of the virus by modifying the coding sequences quickly to adopt challenged rather than non-coding regions.

Further, Iqbal et al.^99^ used CRISPR-Cas9 system to control the begomoviruses, which is a heterogeneous group of virus system. This virus family includes *the Cotton leaf curl Rajasthan virus* (*CLCuRaV), Cotton leaf curl Alabad Virus* (*CLCuAlV), Cotton leaf curl Kokhran vi*rus (*CLCuKoV), Cotton leaf curl Multan virus* (*CLCuMuV*), and *Cotton leaf curl Bangalore virus* (*CLCuBaV*). Synergistically, group of these viruses causes cotton leaf curl disease in cotton plants and possesses a higher risk to yield loss. In this study, the authors proposed that a multiplex type sgRNA that simultaneously might able to edit the genome of the viral complex along with their DNA – satellites to develop resistant cotton plants.^94^ In another study, the genome of *Wheat dwarf virus* (*WDV*) was harshened at four different sites by accessing the activity of four artificially created sgRNA/Cas9 construct and results confirmed that plants were resistant to viral infection, did not show viral symptoms and lost viral activity. Similarly, *endogenous banana streak virus* (*eBSV*) which causes infection in banana plants was inactivated by CRISPR system targeting viral sequences.^95^ The mutation in viral genome led to inactivation of transcriptional/transitional machinery preventing the replication of functional viral proteins thus providing resistance against banana streak disease. These results are of great importance for future research, demonstrating that resistance in plants can be developed by expressing the genes or activating the genes that recognize and target the viral genome and result in viral resistance.

As discussed above, virus relay on plant machinery to imitate the infection, and several receptors in plants serve as S genes that are used by viruses to replicate themselves. Modification/deletion or mutation is S genes is another approach to confer resistance against viruses. However, targeting RNA virus was initially found to be difficult due to the incompatibility of Cas9 (derived from *Streptococcus pyogenes*) to recognize and cut the genome of RNA viruses.^47^ This challenge later led the discovery of new types of Cas nucleases such as *FnCas9* and *LwaCas13a* derived from *Francisella novicida* and *Leptotrichia wadei*, respectively, and was able to trace and bind to viral RNA genome.^28,100^ Following this discovery, resistance against *cucumber mosaic virus* (*CMV*) and *tobacco mosaic virus* (*TMV*) in model plants, i.e.,, *N. benthamiana* and *A. thaliana*, was developed using RNA targeting sgRNA/Cas protein complex. It was found that the resulted transgenic plants have a less viral load (~80%) than the wild type and control plants and the mutation generated using this system were durable to pass up to T_6_ generation.^28,100^ The eukaryotic translation initiation factors such as *eukaryotic translation initiation factor4E* (*eif4e*) and its isoform play an essential role in the plant’s translational course and a decisive susceptible element for viral infection. The study has revealed that loss of function mutation *eIF(iso)4E* gene resulted in *Turnip mosaic virus* (TuMV) resistance without affecting the plant phenotype and overall health.

Further, resistance in crop plants such as cucumber was developed against *Zucchini yellow mosaic virus* (ZYMV), *Papaya ringspot mosaic virus* (PRSMV), and *Cucumber vein yellowing virus* (CVYV) belonging to potyvirus and *ipomovirus* family, respectively. Gomez et al.^93^ targeted the *eif4e* isoforms (novel cap-binding protein-1 and 2, which are associated with viral genome proteins) using CRISPR-Cas9 system and found that mutation in these isoforms reduced the disease symptoms of *Cassava brown streak virus* (*CBSV*). Besides, a mutation in *the translation initiation factor 4 gamma gene* (*eIF4G*) of rice also improved the resistance against *Rice tungro spherical virus* (RTSV) and *Rice tungro bacilliform virus* (RTBV). These two viruses are the causal agent of Rice tungro disease (RTD) and cause severe economic losses.^97^ More recently, *the eIF4E1* gene was targeted using CRISPR-Cas9 and resistance against *the Clover yellow vein virus* (ClYVV) was developed in *A. thaliana*.^101^ The authors of this study reported that a few mutations in *eIF4E* were enough to develop resistance against *ClYVV* without hampering the plant vigor. In other crops such as tomato, melon, strawberry cucumber, etc., also these S genes found to display resistance and hence used as an important target gene to develop resistance in host plants.^102^

## Limitation in Developing Resistance in Plants

In past decade, several studies have shown the potential of CRISPR system in editing the genome of broad range of plant species including monocots, dicots, and increased resistance against number of plant pathogens.^103^ Nevertheless, two-fold limitations are lying there. First is the continually evolving nature of pathogens, which try to modify its genome to break the already available resistance. Also, there is a continuous trade off among plant genes where editing of S gene might cost the plant fitness. The second major limitation is the constraint from CRISPR-Cas9 tools in the form of “off target mutation”. We will be discussing these aspects one by one.

Though knock out a susceptible gene from the host is easy and can be achieved by introducing one or two mutations. Also these mutations are durable also than the R gene mutation because to combat with plant defense system, the pathogen may undertake low selective pressure.^72^ But there is a possibility that this type of loss of mutation in S gene(s) might affect the plant health, growth, and developmental process and help the pathogen to induce infection. Though these mutations might not be lethal but can cause pleiotropic effects such as dwarfness, nutrient deficiency, and suppression of genes involved in replication processes.^16,72^ To address these challenges, it is required to design new S variants^101^ and insert them into the plants, or use of base editing technique or targeting the specific promoter of desired genes/alleles.^104^ However, several studies indicated that even after the deletion or mutation in the translational initiation factor, the plant growth was not affected^86,97^ yet it further needs more experimental pieces of evidence to confirm it.

Off-target mutation is another major limitation of CRISPR-Cas9 system that caught scientist’s attention about its applicability. An off target mutation is an undesired or nonspecific change that occurred in genome in form of point mutation, insertion, and translocations after GE.^43,44^ These undesired mutations can influence the competence of the system or can alter the gene function, structure and may persuade toward cell based on the nature of mutation. However, the off-target mutation in the planta system did not tempt scientist’s attention, considering the thoughts that it can be reversed or removed by backcrosses.^43,44^ Though, it is an issue of concern in reverse genetic studies as well as the generation of some unwanted outputs in the form of superweeds, etc. In recent days, scientists tried to issue the off-target issue either by using computational tools or by re-engineering the CRISPR components such as Cas proteins, gRNA etc. Several bioinformatic tools such as CasOFFinder and CCTop, Guide-seq, HTGTS, BLESS, Digenome-seq and DISCOVER (discussed in,^16^) are used to reduce the risk on off-target mutation. In addition to it, researchers are now using machine learning models such as Bowtie or Elevation, linear regression model, Random Forrest Model to minimize the off-target mutation and improve the efficiency of CRISPR system. Though these models proved effective in several cases but it was found that they work efficiently only with the specific target which was used to create it. So, it is require to develop an advanced model that can work efficiently with border target range for the successful application of the CRISPR-Cas9 system.^105^ More recently, EvolvR- a modified CRISPR-Cas9 tool with an error-prone DNA polymerase system has been developed to modify or introduce nucleotide sequences at specific regions of the genome by gRNAs.^106,107^ On other hand, researchers re-engineered the Cas9 proteins to minimize the off-target mutations. Several Cas9 variants such as eSpCas9, HF-Cas9, HyperCas, and SniperCas9 (details can be found in the article by^16^) were engineered to improve the efficiency and specificity of these proteins. Several other Cas proteins such as NmCas9 (*Neisseria meningitidis*),^108^ SaCas9 (*Staphylococcus aureus*),^109^ StCas9 (*Streptococcus thermophilus*),^110^ FnCas9 (*Francisella novicida*),^98^ and CjCas9 (*Campylobacter jejuni*)^111^ etc., also have been identified and found to be smaller in size than SpCas9. These proteins give advantage as for gene delivery and more specific gene targeting.^112^ Also, the gRNA engineering or CRISPR-Cas9 complex with RNPs was constructed. RNPs are small ribonuclear proteins that have shorter life span and can disintegrate after expressing in the target system. Thus reduce the risk of off-target mutation in the system.^43,44^ Several studies reported the successful application of CRISPR-Cas9/RNP complex. For example, Malnoy et al.^55^ targeted the DIPM genes in apple protoplast using RNP:Cas9 complex and reported that mutation in these genes leads the resistance against fire blight disease. However, plants were not generated from these edited protoplasts. Similarly, rice and Arabidopsis mutants were generated by single and multiplex genome targeting approach and next generation techniques was used to identify the off-target mutation sites and numbers after genome editing. It was noted that this approach is comparatively more specific and produced fewer mutation.^113^

## Ethical Issues with CRISPR-Edited Crops

**Figure 4.**
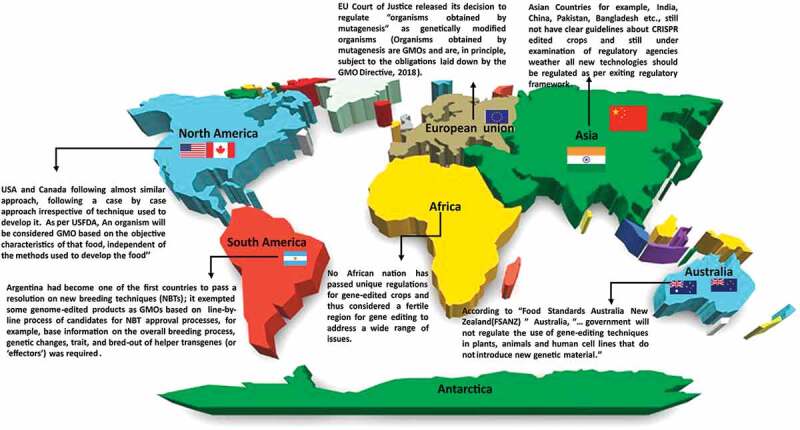
Regulatory approaches around the globe* over CRISPR edited crops and other GMOs

Many studies have reported the efficiency of CRISPR-Cas9 system to the development of disease-resistant transgenic plants. Recently, there was a report of CRISPR-edited tomato plants, which was created using consistently expressing *cas9*, which targets the viral coat protein and replicase genes.^86,97^ These transgenic plants had a remarkably low viral load and were stable up to 3rd generation. However, the application of such constantly expressing nuclease crops comes under the category of genetically modified organisms (GMO); which has to undergo the GMO regulatory measures and not accepted in several countries as well as have a high possibility of off-target mutation. So, it is desirable to develop transgene-free procedures that can employ several pathogen effectors and resistant genes simultaneously, as well as can generate foreign crops that escape the GMO regulations. It is expected that using DNA-free CRISPR-Cas9 systems such as RNP (discussed above) can overcome this limitation and would be helpful in the future. Around the globe, there are different regulatory measures, cultural perceptions, and diverse oversight of CRISPR-edited crops and their acceptance (Fig. 4). National governments worldwide represent different responses to the public opinion and the scientific community and policies made also reflect diverse cultures, environmental conditions, political pressure and interests of farmers, agro-industrial, environmental activists, or agencies. For more details, authors are advised to read the detailed articles over CRISPR policies and regulations written by Globus and Qimron^114^; Friedrichs et al.,^115^; Kawall et al.,^116^ Though CRISPR modified crops with improved phenotypes are being cultivated and sold in a few countries like USA, Canada, still world is fighting to find out if CRISPR crops should be regulated (as GMO) or not. Recently, USA, Canada exempted CRISPR from falling under the definition of a GMO under regulatory regimes and allowed its cultivation and sell without GMO tag. GMO regulation and testing is a tricky process that cost millions of dollars (for field test, data collection, analysis) to release a GMO crop. It also removes the uncertainty of consuming GMO crops among the public. As per the regulations of the United States Department of Agriculture (USDA), CRISPR-Cas9 edited crops can be cultivated and sold free from regulatory monitoring.^117^ To date, CRISPR-Cas9 edited crops have been exempted from GMO regulations; 1. A white button mushroom in which the polyphenol oxidase (PPO) gene is knocked out to develop resistance to browning; 2. Waxy corn in which *the wx1* gene responsible for waxy appearance is inactivated; 3. green bristle grass with delayed flowering time; 4. camelina for improved oil content and; 5. Soybean was tolerant to drought stress.^11,117^

Few countries such as USA, Canada, Aregentina, Australia are having clear and flexible gidelines over the CRISPR edited crops and other GMOs while others such as countries in europe (EU) have more strict move toward it. Some countries like India, China etc., are still in a process to revise the current regulatory guidelines and yet to come over a decision. (*Maps- Not to scale).

## Conclusion and Future Perspectives

Studies discussed here to show the potential of S genes and CRISPR-Cas9 system in developing resistance against several bacterial, fungal, and viral pathogens. CRISPR-Cas9 system is multidimensional, expanded our horizons in the field of genome engineering and enabled us to uncover the exceptional and sophisticated molecular secrets lie within the living system. Still, challenges are there that need to be addressed. But developing resistance against plant pathogens using GE by CRISPR could prove a promising approach to conquer the breeding barriers. Different ‘Omics’ approaches, such as transcriptomics, proteomics, and metabolomics must be utilized to explore the plant defense mechanisms in plant–pathogen interactions studies^118^ The outcome of these studies might provide a border range of cellular targets that can help to generate more resistant plant cultivars. More recently, researchers combined the metabolomics with other “omics” approaches to obtain comprehensive overview of cellular processes in a physiological context.^119^ For example, metabolomics analysis of Soybean hypocotyls, mulberry fruit was performed in response to *Phtyophthora sojae* and *Ciboria shiraiana* infection, respectively ^120^^,121^ and paves the way for future research of critical metabolic determinants in plant–pathogen studies. It is expected that in the future CRISPR system coupled with other techniques (GE/omics) will emerge to create disease-resistant plant varieties that can withstand the environmental, biotic stresses and provide adequate supply of food to the society. The CRISPR-edited crops those are already developed and awaiting to cross the regulatory barriers might also get acceptance and other crops with more desired traits such as improved yield, medicinal properties (in form of edible vaccine) can be developed.

In the end, as a powerful yet versatile gene editing and regulation tool; CRISPR-Cas9 technology is already accelerating each area of science and become the source of sustainable agriculture. We believe its broad applications in plant and microbial biology research will significantly advance our knowledge of both basic biology and disease resistance in the years to come.
